# Gliomas: Survival, origin and early detection

**DOI:** 10.4103/2152-7806.74243

**Published:** 2010-12-25

**Authors:** Patrick J. Kelly

**Affiliations:** New York, New York, U.S.A

**Keywords:** Early detection, glioblastoma, gliomas, stem cells

## INTRODUCTION

I did some of my training with Paul Bucy. He had a special interest in the surgical treatment of glioblastoma. Bucy believed, as did many “cancer surgeons” of his day, that tumors resulted from good cells becoming bad cells that formed a mass of tumor and that cells from the tumor’s periphery invaded the surrounding normal tissue. He correctly observed that malignant gliomas usually grew locally and rarely metastasized outside of the central nervous system. If there was any surgically curable “cancer”, he believed, it was a glioblastoma; all that was necessary for a cure was an aggressive enough resection with an adequate margin.[1]

Of course, his patients died right on schedule, just like anybody else’s patients. Bucy believed that this was because we just could not identify the true margin of the neoplasm at surgery and that the resection was rarely sufficiently adequate to provide a cure.

The advent of computed tomography (CT scanning), magnetic resonance imaging (MRI) and image-guided neuronavigation would change all of this by potentially allowing us to accurately resect as much of a glioma as we chose to resect.

Well, I have spent a career trying to cure gliomas with high technology-based surgery – in particular, imaging-based stereotactically guided volumetric resections – but the long-term survival in the vast majority of these patients is not much better than it was 60 years ago! We just do not hurt these patients as badly as we did 60 years ago. And what do we do? We keep throwing more and more expensive surgical high technology at the problem with marginal improvements in survival, if any.

To be sure, there are some gliomas that we can cure with modern surgical techniques, such as pilocytic astrocytomas, the occasional oligodendroglioma, neurocytomas, gangliogliomas, subependymomas and a few xanthoastrocytomas and protoplasmic astrocytomas. But this is not a credit to neurosurgeons and our modern surgical methods. It is a function of the growth pattern of these particular tumors that lend themselves to complete and curative surgical excision. These tumors have a distinct boundary where tumor stops and normal brain begins. All that a surgeon has to do in these cases is identify the plane between tumor and surrounding brain tissue, develop that plane and remove the tumor. Image guidance helps a bit. But I will point out that Donald Matson claimed a 50% surgical cure rate in pilocytic astrocytomas over 50 years ago – without any “high technology”.

Nonetheless, the “curable” tumors listed above are relatively rare compared to the overwhelmingly more common “fibrillary astrocytomas”, oligodendrogliomas and mixed gliomas. How are we doing with these tumors?

Not so great!

**Figure 1:**
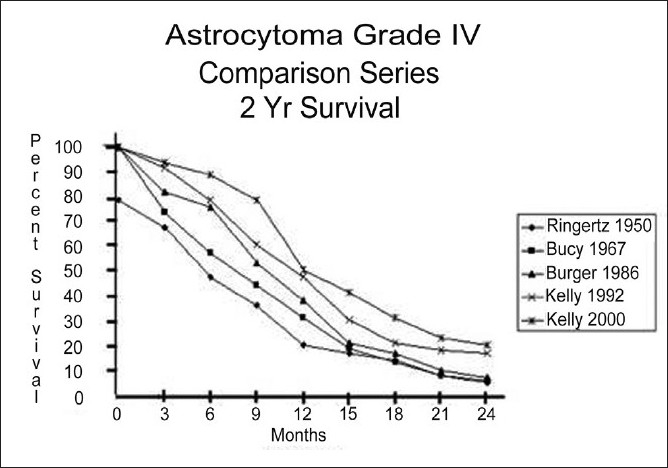
Post surgical survival following resection in patients with grade IV astrocytomas (glioblastoma) in a recent unpublished series (Kelly 2000) compared to survival curves adapted from earlier studies in the literature (Kelly 1992,[3] Burger 1986,[2] Bucy 1987,[4] Ringertz 1950[7]

To be sure, there are many reports in the literature which show that patients having “total resection” and adjuvant therapy do better and live longer than those undergoing biopsy and adjuvant therapy. Comparisons to historical controls attempt to demonstrate the benefit of modern surgical techniques over methods used by past generations. An example of just such an exercise is shown in Figure 1.

These life table survival curves compare my own experience with cases compiled in the years 1992 (published)[3] and 2000 (not published) to the 1986 series published by Burger *et al*,[2] Jelsma and Bucy’s series from 1967[4] and Ringertz’s experience from 1950.[2]

At first glance at Figure 1, it appears that over the years, we have made some progress with better median and 2-year survivals. Regrettably, these experiences are not really comparable for two important reasons. First, survival times are measured from when surgery is performed and histology is available. Modern imaging methods allow patients to be diagnosed much earlier – usually at the onset of the first symptoms – in contrast to patients from the 1950s and 1960s, who could go for weeks or months before a diagnosis was made and surgery performed. Modern series have the benefit of therapy being delivered earlier in the natural history of the disease and a survival starting point that could be weeks or months earlier than in the past decades.

Secondly, modern patients have had the benefit of more effective radiation therapy with linear accelerators instead of cobalt units and more specific chemotherapy. Indeed the improved 2-year survival noted in my 1992 and 2000 series more likely represents the efficacy of carboplatin in the 1980s and temazolamide in the 1990s and not necessarily “better surgery”.

## LOW-GRADE GLIOMAS

**Figure 2:**
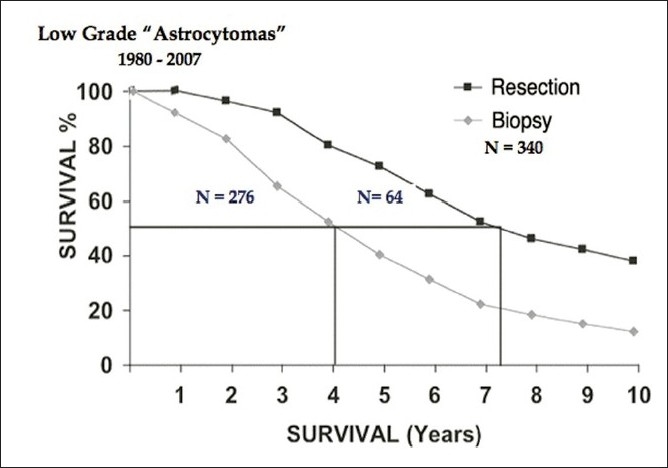
Survival following stereotactic biopsy and stereotactic resection in 340 patients with low grade astrocytomas.

**Figure 3:**
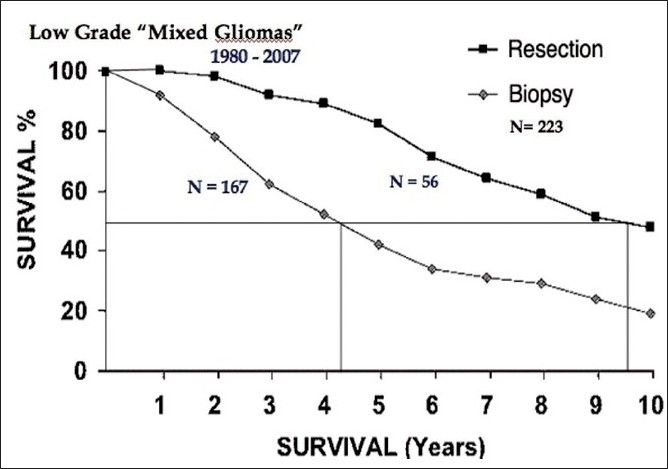
Survival following stereotactic biopsy and stereotactic resection in 223 patients with low grade mixed gliomas.

**Figure 4:**
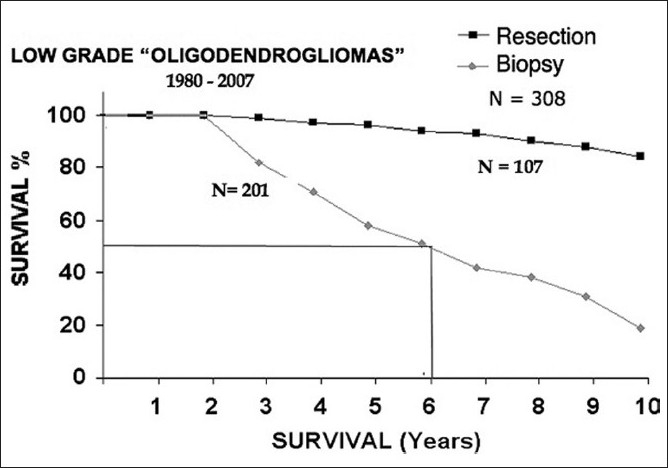
Life table plot of survival following stereotactic biopsy and stereotactic resection in 308 patients with the histologic diagnosis of low grade oligodendroglioma.

In low-grade gliomas, image-guided stereotactic surgical techniques allow us to resect any prospectively designated volume of tissue. Unlike surgery in high-grade gliomas, where we resect a volume of solid tumor tissue and necrosis that has replaced or displaced intact parenchyma and can be resected from just about anywhere in the CNS, resections of low-grade gliomas are restricted by anatomical location. Non-pilocytic astrocytomas, mixed gliomas and most low-grade oligodendrogliomas usually comprise a volume of sick brain tissue infiltrated by isolated tumor cells. Resecting the imaging defined tumor volume is, in fact, resecting intact and functional, albeit “diseased”, brain tissue. Figures 2–4 compare my own experience with stereotactic resection and stereotactic biopsy in low-grade gliomas.

This unpublished series suggests that patients undergoing resection clearly survive longer than those who had only biopsies plus whatever radiation or chemotherapy *de-jour* is administered during the remaining course of their life as their tumors progress from low-grade gliomas to the high-grade tumors that eventually kill them. However, I have never submitted this material for publication for the following two reasons.

### Surgical selection bias

Experienced surgeons usually know which cases will do well with aggressive surgery and which will not. So, relatively compact tumors – especially those in non-eloquent brain areas – will be selected for resection, and diffuse infiltrating tumors in eloquent brain areas will undergo stereotactic biopsy only.

So, studies like this basically compare survival in good surgical candidates to survival in poor surgical candidates rather than comparing the efficacy of aggressive resective surgery to less aggressive surgery. In fact, we may be comparing the natural history of two populations of gliomas, which may have the same histologic cell type but possibly different biologies.

In order to prove the benefit of aggressive resection on survival in gliomas (both high and low grade) we would have to select the “good” surgical candidates and prospectively randomize these into “resection” and “biopsy” groups. However, considering that low-grade gliomas are relatively “rare” and their survival relatively long (in comparison to, say, glioblastoma), a study of this nature would take many years to accrue enough cases with sufficient enough follow-up to justify any conclusions, and would probably require a multicenter effort and a time commitment that would be longer than most academic careers.

### Neuropathology

And so these men of Indostan

Disputed loud and long,

Each in his own opinion

Exceeding stiff and strong,

Though each was partly in the right,

And all were in the wrong!

From The Blind Men and the Elephant; John Godfrey Saxe; 1873

Many neurosurgeons and neuroncologists would like to believe that low-grade gliomas fall into clear-cut histologically homogeneous groups: astrocytomas, oligodendrogliomas and mixed gliomas (*oligodroastrocytoma* or *astro-oligodendroglioma*). Most, however, do not. Serial stereotactic biopsy studies frequently show geographic phenotypic heterogeneity in the predominant cell type within individual tumors. In addition, review of surgical specimens by different neuropathologists usually results in different histologic diagnoses on the same surgical specimen! Some focus on the number of cells that stain positive with Glial fibrillary acidic protein (GFAP) and ignore other cells; others note the many oligodendroglial cells and feel that the many astrocytes are astrogliotic and most discount a neuronal component. I have had low-grade glioma surgical specimens reviewed by as many as eight different well-respected neuropathologists and received as many as eight different histologic diagnoses as regards cell type and grade. Diagnostic interobserver variability between neuropathologists in establishing predominant cell type and grade in gliomas in general and low-grade gliomas, in particular, is a well-recognized problem. This makes a mockery of cell type stratification in low-grade glioma follow-up studies.

It is not the intention to impugn neuropathologists here; they are the true scholars and intellectuals in our field. Their reviews of surgical specimens are usually thorough and their conclusions well considered. But why cannot they agree? The reason, in the review of a glioma specimen, is that they are all right and, in many cases, they are wrong – like the blind men and the elephant. Many of us are fixated in the Cushing-Bailey concept of glial tumorigenesis that tumors developed from dedifferentiated mature cell lines. Astrocytomas resulted from the dedifferentiation of mature astrocytes; oligodendrogliomas from oligodendocytes, etc. At least that was the old way of thinking. Few believe this anymore.

## WHERE DO GLIOMAS COME FROM?

**Figure 5:**
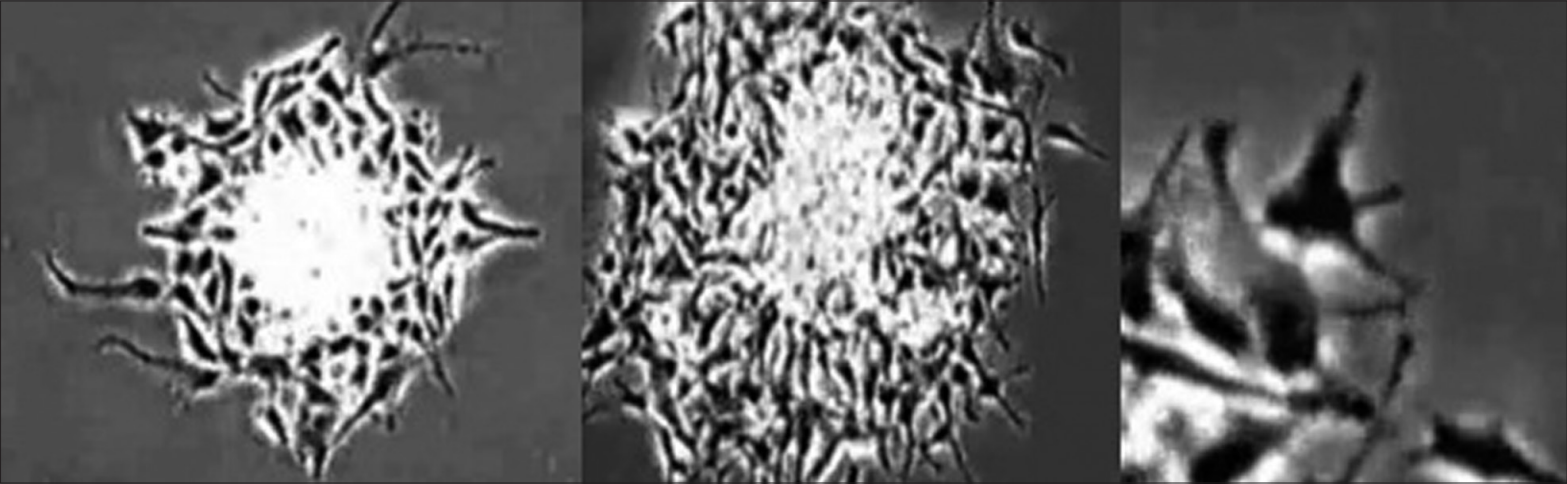
Three frames of time-lapse movie of micro-organ culture of portion of stereotactic biopsy obtained from tissue surrounding a glioma mass. Note amoeboid cells with pseudopods used for propulsion.

A more plausible theory is that all gliomas start out as mixed gliomas. All start out their glioma life containing cells with astrocytic, oligodendroglial and neuronal phenotypes. Over time, the phenotypic clone with the highest mitotic rate becomes the predominant cell type. Gliomas probably evolve from stem cells and the lineage specific progenitors that form neurons, astrocytes and oligodendrocytes. Of course, there remains the possibility that mature differentiated cells revert to progenitor or stem cell status from which a glioma evolves. Nonetheless, examination of a young glioma will reveal GFAP positive cells (astrocytes), synaptophysin positive cells (primitive neurons), cells that stain with neither GFAP nor synaptophysin and are probably oligodendrocytes and perhaps even cells that stain with CD133 that is supposed to identify stem cells. Furthermore, microscopic examination of specimens obtained from the periphery of glial tumors at stereotactic serial biopsy procedures show isolated cells in the extracellular spaces within otherwise normal parenchyma. Time-lapse photography of cell cultures containing tissue from these biopsy specimens demonstrates amoeboid-like cells. They move by pseudopod propulsion – like stem cells which are also motile and also move by pseudopod propulsion [Figure 5].

In normal organogenesis, cytokines bind to notch receptors that cause the stem cell to become a lineage specific progenitor which then progresses to the more specialized cells of the CNS: neurons, oligodendrocytes and astrocytes. In the developing nervous system, neurons need astrocytes, oligodendrocytes and a blood supply from endothelial cells that form capillaries. They signal this need to stem cells by growth factors (cytokines) that bind in the notch receptors of their cellular membrane which begins the intercellular cascade that transforms stem cells into the specialized cells required.

All of this is fine in the developing nervous system, but what happens after the brain has developed?

There are stem cells left over; what happens to them? Others retire to stem cell clusters of 50–100 cells that self-renew and die. Some are probably called up for brain repair in the case of injury or to simply maintain failing cells in the mature brain. Specific growth factors may provide a “call to action”. Or perhaps, these stem cells leave the cluster on their own and wander through the extracellular spaces of the white matter and neuropil. Somewhere, some stop wandering, start reproducing and form neural, oligodendroglia and astrocytic progenitors that also reproduce and this is the genesis of a glioma that contains astrocytic, oligodendroglial and neuronal phenotype – a “mixed glioma”.

All cells have a cell cycle. New cells are born (mitosis) and others die (apoptosis). Undoubtedly, many nascent “tumors” reach a “steady state” where the mitotic rate equals the apoptotic rate and the tumor never grows, never becomes symptomatic and simply exists as a heterogeneous collection of cells co-existing with normal cells in the extracellular fluid of the interstitial spaces. However, occasionally, the mitotic rate exceeds the apoptotic rate and the early tumor begins to add cells. As their numbers increase, their metabolic by-products increase the local osmotic gradient of the extracellular fluid. This results in the ingress of fluid from the intravascular space, and if the spatial volume of this process is large enough, an MRI may now detect a small region of T2 prolongation.

The mitotic and apototic rates differ in the neuronal, astrocytic and oligodendroglial populations of these young gliomas. The cellular clone with the highest mitotic and lowest apoptotic rates will eventually become the dominant cell type. Thus, mixed gliomas containing primitive neurons, astrocytes and oligodendrocytes will eventually become whatever phenotype has the highest mitotic and lowest apoptotic rate as those phenotypic cells can eventually overwhelm and replace the other two cell types in the neoplasm.

The vast majority of gliomas may start out as mixed gliomas having neuronal, astrocytic and oligodendroglial components, but with sufficient time, cellular clones with the highest mitotic rate become the dominant phenotypic cell type from which offspring with even higher mitotic rates evolve.

Low-grade mixed gliomas become high-grade gliomas with a predominant cell type that ultimately kills the patient. The transition from low-grade mixed glioma to malignant glioma of a single cell type, e.g. glioblastoma, malignant oligodendroglioma or very rarely a malignant neurocytoma can occur in weeks, months or many years. Also, it is possible that some early gliomas reach a steady state in which a low mitotic rate is matched by a similar apoptotic rate and the lesion never progresses or possibly even regresses over time.

## GLIOMAS ARE MORE COMMON THAN PREVIOUSLY THOUGHT

In the USA, about 19,500 new gliomas are diagnosed each year. Considering that the US population in 2008 was 306 million people, this works out to a glioma incidence rate of 0.00064. That is, 0.0064% or 6.4 cases per 100,000 population. There are some data showing that the incidence of gliomas in the US has increased slightly in the recent years, probably because we are finding more of them due to increased awareness and the easy availability of diagnostic imaging. However, this is just the tip of the iceberg; the actual number of gliomas in the USA is probably 40–50 times greater than the reported incidence.

Over the years, there have been about 16 reported studies focusing on the incidence of CNS disease detected by CT and MRI within the “normal” asymptomatic population.[6] These have reported various incidences of supposed gliomas ranging from 0 to 6 gliomas per thousand population. A more conservative recent study of 1000 asymptomatic volunteers, conducted by the NIH and reported in the *Journal of the American Medical Association (JAMA)*, found three gliomas in those 1000 individuals.[5]

Extrapolating to the entire population of the USA, three in a thousand works out to over 900 thousand Americans harboring asymptomatic gliomas! Only a small number of these will become symptomatic, diagnosed and recorded in any given year. We know that number, which is about 19,500. We do not know how many of these will become symptomatic, diagnosed and treated in a lifetime.

Also, there is the possibility that many more people may harbor microscopic gliomas that have not yet sufficiently influenced the parenchymal interstitial microenvironment to be detectable by MRI.

Nonetheless, those 900 thousand or so individuals may represent a “population at risk” for developing a symptomatic, and most likely incurable, glioma in their lifetime even though only a small percentage becomes symptomatic in any given year.

## WHY WE CANNOT CURE GLIOMAS

By the time a glioma becomes symptomatic, it is almost always too late in its biological course. Motile isolated tumor/stem cells would have migrated far beyond the imaging-defined tumor mass. These will ultimately start another tumor nidus in the margin of the resection, at some distance from the margin or, indeed, even in the opposite hemisphere. Also, this is purely a function of time, with or without the benefit of surgery.

Radiation therapy and chemotherapy may have some effect on some of these cells but there will always be individual cells or even a small population of cells that will not be affected by these modalities – just like the normal cells of the brain that, we hope, are not affected by treatment.

The real culprits are not necessarily the “cancer” cells. The real culprits are the cells that are mostly like “normal” cells – the stem cells. By the time a glioma is diagnosed – by the time it becomes symptomatic and an imaging study is performed – the vast majority are incurable.

Some neurosurgeons might recall a case or two which presented with an MRI showing a significant glioblastoma but also happened to have had an earlier MRI done for some other reason, a headache or minor trauma, etc., and the earlier MRI being perfectly normal. I have had a few cases like this also. I submit that in these cases the transition from low-grade mixed glioma to, say, malignant astrocytoma occurred much more rapidly than most – over a few weeks or months, perhaps. I believe that such cases are relatively rare. In fact, I have seen many more cases where physicians have watched a T2 abnormality on MRI getting larger and larger over several years but feel that they must wait for symptoms before recommending surgery. This reminds me of my friend Thor Sundt’s joke about the man jumping off the top of the Empire State Building, passing someone on the 42^nd^ floor who calls out: “How are you doing?”

The Jumper calls back: “I’m doing fine so far!”

## SCREENING FOR GLIOMAS

Many studies have shown that current therapies (surgery, radiation, chemotherapy, etc.) expand survival in gliomas beyond the natural history of the disease. However, in the vast majority of cases, gliomas are ultimately incurable. Most low-grade gliomas kill patients by becoming high-grade gliomas. Even low-grade gliomas are incurable because by the time they are diagnosed, the disease process has extended far beyond the limits of surgical resectability. Also, no treatment that I know of will prevent a low-grade glioma from ultimately becoming a high-grade glioma – except, perhaps, surgical total excision of a small low-grade lesion.

In my opinion, gliomas are incurable because we are finding them far too late in their clinical course. It is like finding breast cancer after it has spread to the regional nodes, lung, liver, skeletal system or brain, prostate cancer after it has spread to the pelvis and spine, colon cancer after it has metastasized to the liver, skin melanoma after it has spread to lymph nodes and beyond, etc. However, there are screening programs for the early detection of all of these “cancers”: self-examination and mammography for breast cancer, PSA blood tests for prostate cancer, colonoscopy for colon cancer, dermatological examinations for skin cancer, etc. Why not screen for brain tumors?

Unlike these other “cancers”, brain tumors grow by local invasion. Brain tumors rarely metastasize outside of the central nervous system. If the concept of early detection has any merit at all, it should be in the early detection of gliomas: find them when they are small, find them before they turn malignant and find them when they may still be curable by some minimally invasive surgical method or even by stereotactic radiation methods such as brachytherapy or radiosurgery.

In addition, it is much easier and safer to operate on a small lesion, be it a glioma, meningioma, acoustic neurinoma or whatever, than a big one!

In the 1950s, clinics and mobile X-ray units offered free or low-cost screening chest X-rays for the early detection of tuberculosis. This was probably one of the most effective public screening programs ever. Early detection of pre-clinical disease and isoniazid wiped out tuberculosis in the USA in a few years. What screening tools are available for gliomas?

Perhaps, a blood test and genetic screening for brain tumors may be possible some day. However, since 1973, we have had an excellent tool for brain tumor screening, i.e. MRI. And what do we use it for? We use it to make a diagnosis in symptomatic patients who, by the time they are diagnosed, have essentially an incurable disease! Why not use MRI to screen for brain tumors in an early detection program? We screen for other tumors; why not brain tumors?

Radiologists who are used to mammograms and chest X-rays usually raise the issue of “false positives.” But unlike X-ray based procedures, MRI provides various imaging sequences to non-invasively investigate abnormalities. In particular, MR spectoscopy (MRS) is very useful in the determining whether a unidentified bright object (UBO) is a glioma instead of a demyelinating plaque, microinfarction or some other non-neoplastic process. However, what do we do when an abnormality suspicious for a glioma is found?

As we have seen above, the clinical incidence of gliomas is orders of magnitude lower than the assumed prevalence in an asymptomatic population. It is possible that many incidentally found gliomas will never grow. Indeed, some smaller lesions may even regress. Most may never require treatment in the near future. Some may never need treatment.

Those with an MRI defined abnormality, in whom MRS suggested glioma, would represent a “population at risk”. These would require follow-up imaging. Treatment would be recommended for those having lesions larger than, say, 2 cm in diameter or those in whom documented growth or change in a small lesion is noted on follow-up imaging or the lesion becomes symptomatic.

Many point out that screening is not “cost effective”. I agree. It is certainly less expensive to treat a small number of afflicted people with ineffective and expensive therapies than it is to screen a large healthy population. But this argument could be made for screening, in general. Nonetheless, a screening MRI for the detection of early gliomas only requires two or three imaging sequences (T1, T2 and FLAIR). Contrast enhancement would not be necessary – it would be a very rare tumor that would exhibit contrast enhancement and not show an abnormality on T2 or Flair images. The cost of a diagnostic MRI with multiple sequences and contrast enhancement in New York City, at least, is about $1000 and the entire examination takes about 45–60 minutes. A screening MRI would require about 3–5 minutes scanning time, and as a proportion of the cost of a diagnostic examination, should cost only $60–80 which would compare favorably to the cost of a colonoscopy (average: $2000–3734), total PSA blood test (between $70 and 400), mammography ($140–320), skin screening (about $150 plus), etc. Of course, someone would have to read the MRIs but computer-assisted diagnosis (CAD) systems should reduce the tedium and costs.

Over the past 30 years, we have seen real progress in the development of sophisticated surgical technology. Computer-based medical imaging combined with stereotactic navigation techniques for minimally invasive surgical or non-invasive radiosurgical methods, intraoperative imaging, mapping procedures, etc., all of these combine to make tumor neurosurgery less invasive, more effective and safer. However, in the resection of gliomas, we are fighting a war that would be easier and more likely to win if we begin before the enemy becomes extensive and well entrenched in the “civilian population”. We need a screening program for the early detection of gliomas.

## AN EARLY-DETECTION PILOT PROJECT

**Figure 6:**
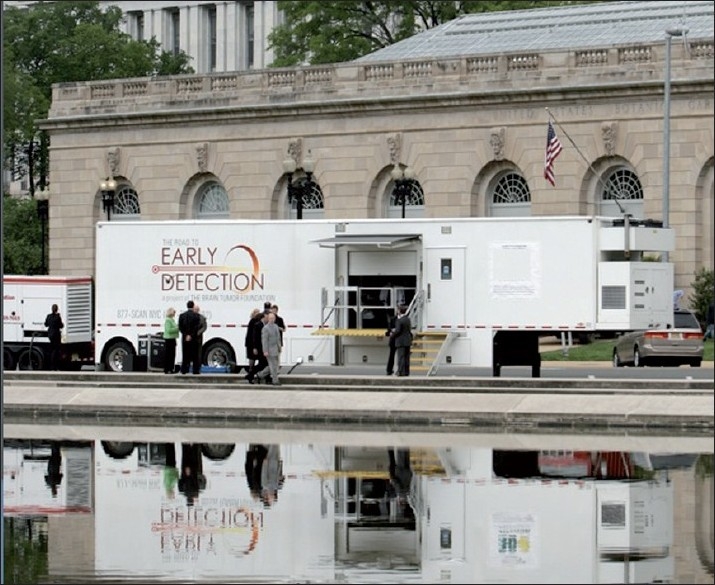
Mobile MRI unit at the reflecting pool in Washington, DC offering scans to members of the U.S Congress and their staffs. Results? Classified.

About two years ago, the Manhattan based Brain Tumor Foundation began such a program in New York City (http://www.roadtoearlydetection.org). A General Electric 1.5 Tesla MRI unit, housed in a truck, makes the rounds of the five boroughs of New York City offering free screening head MRI scans to anybody who wants one [see Figure 6]. The response from the general public has been overwhelmingly positive. (The response from the local medical profession has been, predictably, lukewarm to downright hostile.) This project is supported by public funds and private donations. Data, collected prospectively, will be analyzed in collaboration with the Department of Epidemiology at Columbia University.
